# Supplementary material for: Ultrasound-Assisted Extraction of Applewood Polyphenols at Lab and Pilot Scales
Source: Foods. 2023 Aug 22;12(17):3142. doi: 10.3390/foods12173142 (PMC10486881; doi:10.3390/foods12173142)
Supplement: Supplementary file 1 [file foods-12-03142-s001.zip › foods-2504569-supplementary.pdf]

Table 1 S: HPLC calibration parameters of phenolic compound standards

**Compound**	**Retention time** (min)	**Wavelength** (nm)	**Linear range** (mg/L)	**Slope** (mAU.L/mg)	**Intercept** (mAU)	**Correlation coefficient** (R^2)	**Limit of detection** (mg/L)	**Limit of quantification** (mg/L)
Gallic acid	5.49	260	1.00 – 50.2	0.02168	-0.01041	0.9998	0.076	0.25
Procyanidin B1	9.07	280	1.00 – 50.0	0.006338	-0.003927	0.9991	0.086	0.29
(+)-Catechin	12.04	280	2.00 - 100	0.006072	-0.001217	0.9999	0.045	0.15
Procyanidin B2	13.43	280	1.00 – 50.0	0.005306	-0.002393	0.9994	0.048	0.16
Chlorogenic acid	15.79	320	0.99 – 49.4	0.02538	-0.001921	0.9999	0.050	0.17
Vanillic acid	16.63	260	1.02 – 51.0	0.03216	-0.004669	0.9999	0.070	0.23
(-)-Epicatechin	18.20	280	0.98 – 49.2	0.005975	-0.003548	0.9998	0.80	2.65
p-Coumaric acid	24.24	290	1.00 – 50.2	0.06049	-0.009602	0.9999	0.029	0.098
Epicatechin gallate	24.37	280	1.11 – 55.5	0.01550	-0.008321	0.9997	0.20	0.66
Naringin	34.13	280	1.07 – 53.4	0.01469	-0.002246	0.9999	0.067	0.22
Quercetin 3-D-galactoside	35.73	260	1.00 – 50.0	0.05722	-0.009052	0.9999	0.039	0.13
Phloridzin	38.00	280	2.01 – 101	0.01800	-0.003294	0.9999	0.023	0.078
Avicularin	40.29	260	1.00 – 50.0	0.02438	-0.004669	0.9999	0.048	0.16
Kaempherol 3-O-glucoside	41.88	260	1.00 – 50.0	0.02372	-0.003494	0.9999	0.030	0.10
t-Cinnamic acid	43.03	280	0.49 – 24.6	0.07593	-0.005454	0.9999	0.039	0.13
Naringenin	47.97	290	0.80 – 40.0	0.008192	-0.001389	0.9999	0.035	0.12
Quercetin	48.70	360	1.87 – 93.4	0.02998	-0.02244	0.9998	0.19	0.62
Phloretin	50.45	280	0.90 – 45.2	0.03245	-0.004946	0.9999	0.056	0.18
Daidzein	44.32	260	ISTD*	-	-	-	-	-

*ISTD = Internal Standard
